# Mediastinal Lymphadenopathy Diagnosed as Prostate Cancer via Endobronchial Ultrasound-Guided Transbronchial Needle Aspiration (EBUS-TBNA) in a Patient With a History of Renal Cell Carcinoma: A Case Report

**DOI:** 10.7759/cureus.79446

**Published:** 2025-02-22

**Authors:** Naoaki Tsuji, Hirai Soichi, Kano Yukari, Tanimura Mai, Shiotsu Shinsuke

**Affiliations:** 1 Pulmonology, Kyoto Prefectural University of Medicine, Kyoto, JPN; 2 Pulmonology, Japanese Red Cross Kyoto Daini Hospital, Kyoto, JPN

**Keywords:** ebus-tbna, immunohistochemistry, mediastinal lymphadenopathy, prostate cancer metastasis, renal cell carcinoma

## Abstract

In individuals with a history of renal cell carcinoma (RCC), mediastinal lymphadenopathy is frequently attributed to metastatic recurrence. However, secondary malignancies, despite their rarity, should also be considered. During routine follow-up examinations, a 63-year-old male with a history of renal cell carcinoma demonstrated progressive mediastinal lymphadenopathy. This was initially suspected to be a recurrence of renal cell carcinoma (RCC). The diagnosis of metastatic prostate adenocarcinoma was confirmed by prostate biopsy and elevated prostate-specific antigen (PSA) levels, as determined by endobronchial ultrasound-guided transbronchial needle aspiration (EBUS-TBNA). Treatment with androgen deprivation therapy (ADT) and chemotherapy was initiated after the patient was diagnosed with metastatic prostate cancer. This case underscores the critical role of EBUS-TBNA in the accurate diagnosis of patients with a history of RCC who demonstrate atypical mediastinal lymphadenopathy. The provision of tissue samples for histopathological analysis by EBUS-TBNA facilitated the differentiation between metastatic recurrence and secondary malignancies. Comprehensive diagnostic methods, including tumor marker analysis, immunohistochemistry, and tissue biopsy, are essential for accurate diagnosis. EBUS-TBNA is indispensable for accurate diagnosis, especially in differentiating metastatic recurrence from secondary malignancies, as it provides a minimally invasive method of tissue access for precise evaluation.

## Introduction

A 63-year-old man with a history of renal cell carcinoma (RCC) underwent routine follow-up imaging, which revealed progressive enlargement of mediastinal lymph nodes. Although other potential causes, such as lymphoma or sarcoidosis, were considered, these were deemed less likely based on blood test results and clinical evaluation. Consequently, metastatic RCC was suspected. Further diagnostic workup, including endobronchial ultrasound-guided transbronchial needle aspiration (EBUS-TBNA), led to the unexpected diagnosis of prostate adenocarcinoma. This case highlights the diagnostic challenges in distinguishing between the metastatic recurrence of a known malignancy and the manifestation of an undiagnosed secondary primary cancer.

Mediastinal lymphadenopathy in patients with a history of RCC is commonly attributed to metastasis, particularly in cases of unusual or isolated patterns of nodal involvement [1]. However, such presentations warrant a thorough evaluation, as secondary malignancies, though rare, can mimic the recurrence of the primary tumor [2]. In this patient, the absence of typical RCC metastatic patterns and the atypical site of metastasis prompted a detailed investigation. Immunohistochemistry and serum tumor marker analysis, particularly prostate-specific antigen (PSA), played a pivotal role in confirming the diagnosis of prostate cancer.

We report a rare case of mediastinal lymph node metastasis as the initial manifestation of previously undiagnosed prostate cancer in a patient with a history of RCC. This case underscores the importance of comprehensive diagnostic approaches in evaluating metastatic lesions in patients with a history of malignancy, as misdiagnosis may lead to inappropriate management.

## Case presentation

**Figure 1:**
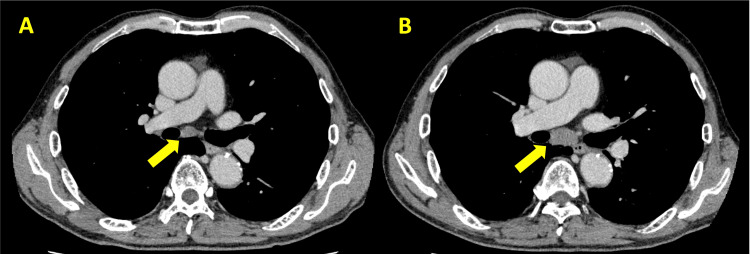
Progressive enlargement of mediastinal lymph nodes on contrast-enhanced CT A: initial axial contrast-enhanced computed tomography (CT) image of the chest showing mild mediastinal lymphadenopathy (yellow arrow) without significant compression of adjacent structures; B: follow-up axial contrast-enhanced CT image of the chest demonstrating progressive enlargement of the mediastinal lymph node (yellow arrow) compared to image (A). This increase in size raised suspicion of malignancy, leading to further evaluation via EBUS-TBNA. EBUS-TBNA: endobronchial ultrasound-guided transbronchial needle aspiration

A 63-year-old man with a history of left partial nephrectomy for left renal cell carcinoma (RCC) four years earlier presented for routine follow-up imaging. He was initially diagnosed with RCC and underwent nephron-sparing surgery at that time. Pathological examination of the resected specimen revealed clear cell renal cell carcinoma (ccRCC), classified as pT1aN0M0, Ly0, V0, Grade 2 (World Health Organization (WHO)/International Society of Urological Pathology (ISUP) grading system). Postoperative imaging and follow-up were unremarkable until routine surveillance revealed mediastinal lymphadenopathy. The patient had a 20-pack-year smoking history but had quit smoking three years prior. He reported no systemic symptoms such as fever, night sweats, weight loss, or respiratory complaints. Computed tomography (CT) showed progressively enlarging mediastinal lymph nodes without evidence of pulmonary involvement or other distant metastases. Figure 1 illustrates the contrast-enhanced CT images demonstrating the progression of mediastinal lymphadenopathy over time.

Differential diagnoses included lymphoma, sarcoidosis, and tuberculous lymphadenitis; however, blood tests, including interleukin-2 receptor (IL-2R), angiotensin-converting enzyme (ACE), and QuantiFERON-TB Gold (QFT), were negative. Table 1 presents the general blood test results.

**Table 1:** Blood test results This table summarizes the patient's laboratory results, including hematologic parameters, renal and hepatic function tests, inflammatory markers, and tumor markers. Notably, lung cancer tumor markers were included to ensure a comprehensive oncologic evaluation. PT: prothrombin time; INR: international normalized ratio; APTT: activated partial thromboplastin time

Test	Result	Normal range
White blood count (WBC)	6.8×10³/uL	4.00 to 11.00 ×10³/uL
Hemoglobin (Hb)	13.8 g/dL	12 to 16 g/dL
Platelet	245×10³/uL	150 to 450×10³/uL
Serum creatinine	0.80 mg/dL	0.5 to 1.1 mg/dL
Serum sodium	141 mmol/L	136 to 145 mmol/L
Serum potassium	3.4 mmol/L	3.5 to 5.0 mmol/L
Total protein	8.1 gm/dL	6.4 to 8.3 gm/dL
Lactate dehydrogenase (LDH)	189 IU/L	135 to 250 IU/L
C-reactive protein (CRP)	0.33 mg/dL	0 to 5 mg/dL
Carcinoembryonic antigen (CEA)	9.5 ng/mL	0 to 5 ng/mL
Pro-gastrin-releasing peptide (proGRP)	41.1 pg/mL	0 to 80.9 pg/mL
Cytokeratin 19 fragment (CYFRA 21-1)	1.3 ng/mL	0 to 3.3 ng/mL
Interleukin-2 receptor (IL-2R)	391 U/mL	122 to 496 U/mL
Aspartate aminotransferase (AST)	13.3 U/L	10 to 40 U/L
Alanine aminotransferase (ALT)	10.6 U/L	5 to 45 U/L
PT (%)	105.7%	70 to 130%
PT-INR	0.97	0.8 to 1.2
APTT	27.8 sec	24 to 34 sec
D-dimer	0.5 mg/L	<1.0 mg/L

**Figure 2:**
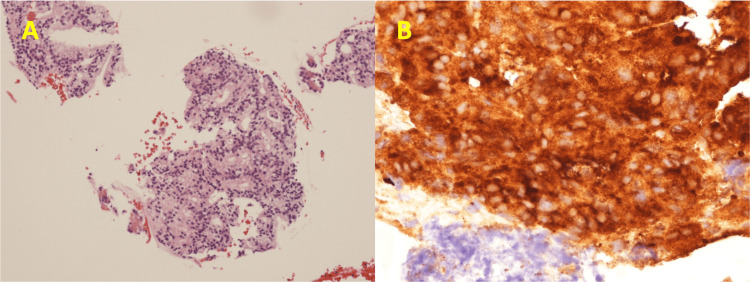
Histopathological and immunohistochemical findings of mediastinal lymph node specimen A: Hematoxylin and eosin (H&E) staining of the mediastinal lymph node tissue specimen obtained by EBUS-TBNA, showing fused glandular proliferation of cells with round nuclei and pale eosinophilic cytoplasm. The glandular formation is observed within some areas of the cell clusters. H&E stain, ×100. B: Immunohistochemical staining of the same specimen reveals strong positivity for prostate-specific antigen (PSA), suggesting the presence of metastatic prostate cancer. EBUS-TBNA: endobronchial ultrasound-guided transbronchial needle aspiration

Considering the patient’s history of malignancy and the overall clinical context, metastatic RCC was deemed a likely cause. To confirm this suspicion, EBUS-TBNA of the mediastinal lymph nodes was performed. Histological analysis revealed fused glandular proliferation of cells with round nuclei, pale cytoplasm, and mild nuclear atypia. The findings of typical ccRCC, such as clear cytoplasm and a delicate fibrovascular network surrounding each tumor nest, were not observed. Immunohistochemical staining showed tumor cells were negative for PAX8 and TTF1 and strongly positive for prostate-specific antigen (PSA). Figure 2 shows the EBUS-TBNA specimen with hematoxylin and eosin staining (A) demonstrating glandular proliferation and immunohistochemical staining (B) revealing PSA positivity.

**Figure 3:**
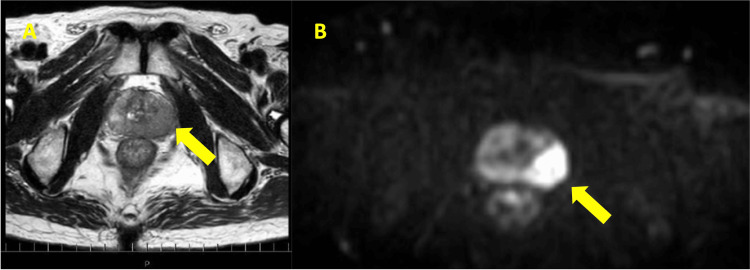
Pelvic MRI showing PI-RADS score 5 lesion A: Axial T2-weighted imaging (T2WI) of the pelvis demonstrates a hypointense signal lesion in the left lobe of the prostate (yellow arrow), suggestive of prostate cancer. The lesion measures approximately 20 mm and is primarily located in the peripheral zone of the left lobe. The extracapsular extension was suspected; however, there was no definitive evidence of seminal vesicle invasion. B: Diffusion-weighted imaging (DWI) reveals a large area of diffusion restriction in the peripheral zone of the left lobe (yellow arrow), further supporting the suspicion of malignancy. PI-RADS: Prostate Imaging Reporting and Data System

Based on the histological and immunohistochemical findings, the likelihood of metastatic RCC and metastatic lung adenocarcinoma was significantly reduced, and metastatic prostate cancer was suspected. Serum PSA levels were then measured and found to be markedly elevated at 351 ng/mL. This prompted further evaluation, including a transrectal prostate biopsy, which confirmed the diagnosis of previously undiagnosed prostate adenocarcinoma with a Gleason score of 4+5=9. The patient was diagnosed with metastatic prostate cancer involving the mediastinal lymph nodes. A pelvic MRI revealed the primary tumor in the prostate but showed no definitive metastatic lesions elsewhere. Figure 3 shows the pelvic MRI, demonstrating a hypointense signal in the left lobe of the prostate on T2-weighted imaging (T2WI).

Contrast-enhanced CT of the chest, performed prior to EBUS-TBNA, showed no evidence of metastasis outside the mediastinal lymph nodes. A multidisciplinary team initiated androgen deprivation therapy (ADT) combined with chemotherapy as the first-line treatment. Based on imaging and histological findings, the clinical stage was determined as cT3aN1M1c (American Joint Committee on Cancer (AJCC) 8th edition), indicating extracapsular extension and mediastinal lymph node metastasis.

This case underscores the importance of a systematic diagnostic approach in patients with a history of malignancy presenting with atypical metastatic patterns. The integration of EBUS-TBNA, immunohistochemical analysis, and tumor marker evaluation was pivotal in identifying the true etiology of the mediastinal lymphadenopathy and avoiding misdiagnosis as RCC recurrence.

## Discussion

The differential diagnosis of mediastinal lymphadenopathy in patients with a history of renal cell carcinoma is challenging, as it is often attributed to metastatic recurrence [1,3]. However, metastasis from secondary primary malignancies must also be considered, particularly when the pattern of metastasis is atypical. In this case, mediastinal lymphadenopathy without lung involvement was inconsistent with the typical metastatic spread of RCC, prompting further investigation [2,4].

EBUS-TBNA proved crucial in obtaining tissue samples for histopathological analysis. The identification of prostate-specific markers, including PSA, provided critical evidence pointing to an undiagnosed primary prostate cancer [5]. The subsequent detection of significantly elevated serum PSA levels and confirmation by prostate biopsy established the diagnosis of prostate adenocarcinoma with mediastinal lymph node metastasis. This highlights the importance of integrating tissue biopsy, immunohistochemistry, and tumor marker analysis for accurate diagnosis in such cases.

Metastatic prostate cancer often involves bones and regional lymph nodes, and isolated mediastinal lymphadenopathy is rare [6]. The pathway of metastasis to mediastinal lymph nodes in prostate cancer may involve retroperitoneal lymphatic drainage or hematogenous dissemination [7]. This case adds to the limited body of literature describing such atypical presentations and underscores the necessity of maintaining a broad differential diagnosis when evaluating metastatic lesions.

Failure to recognize secondary primary malignancies may lead to misdiagnosis and inappropriate management. In this case, misattributing the mediastinal lymphadenopathy to RCC recurrence could have resulted in unnecessary surgical or systemic treatment specific to RCC. Instead, the accurate identification of metastatic prostate cancer allowed the initiation of androgen deprivation therapy and chemotherapy, the standard treatment for advanced prostate cancer.

This case also highlights the evolving role of EBUS-TBNA as a minimally invasive diagnostic tool for mediastinal lesions. In patients with a history of malignancy, it provides a safe and effective means to obtain diagnostic tissue samples, reducing the need for more invasive procedures such as mediastinoscopy [8]. Furthermore, the integration of tumor markers such as PSA offers additional diagnostic precision, especially when the primary malignancy is not immediately apparent.

In conclusion, this case illustrates the importance of a systematic diagnostic approach in evaluating mediastinal lymphadenopathy in patients with a history of malignancy. The combination of imaging, tissue biopsy, immunohistochemistry, and tumor marker analysis was pivotal in identifying metastatic prostate cancer, a rare and unexpected cause of mediastinal lymphadenopathy in this clinical setting. This diagnostic strategy not only avoids misdiagnosis but also ensures appropriate and timely treatment for the patient.

## Conclusions

This case illustrates the importance of comprehensive diagnostic approaches in evaluating mediastinal lymphadenopathy in patients with a history of malignancy. EBUS-TBNA was pivotal in obtaining tissue samples for histopathological and immunohistochemical analysis, enabling the accurate diagnosis of metastatic prostate cancer. This minimally invasive approach reduced the need for more invasive diagnostic procedures while facilitating the timely initiation of appropriate treatment. Accurate diagnosis not only avoided mismanagement but also ensured effective therapy tailored to the patient’s specific condition.
